# Reviewer acknowledgement 2013

**DOI:** 10.1186/1471-2180-14-20

**Published:** 2014-01-29

**Authors:** Philippa K Harris

**Affiliations:** 1 BioMed Central, 236 Gray's Inn Road, London WC1X 8HB, UK

## Abstract

**Contributing reviewers:**

The editors of BMC Microbiology would like to thank all our reviewers who have contributed to the journal in Volume 13 (2013).

## 

Sophie Abby

France

Allison Abplanalp

USA

Florence Abra

Ireland

Analia Abraham

Argentina

Jacqueline Abranches

USA

Tomabu Adjobimey

Germany

Sina Adrangi

Iran

Anton Aebischer

Germany

Claudio Aguilar

Switzerland

Niyaz Ahmed

India

Jiyoung Ahn

USA

Joong-Hoon Ahn

South Korea

Takuya Aikawa

Japan

Kazuya Akimitsu

Japan

Leclercq Alexandre

France

Adriana Alippi

Argentina

Heather Allen

USA

Kevin Allen

Canada

Michael Allen

USA

Emma Allen-Vercoe

Canada

Franz Allerberger

Austria

Francis Alonzo

USA

Adela Alvarez-Buylla

Spain

Sergio Alvarez-Perez

Spain

Masashi Amano

Japan

Ayachi Ammar

Algeria

Hany Anany

Canada

Birgitte Andersen

Denmark

Deborah Anderson

USA

Robin Anderson

USA

Chris Anthony

UK

Josefa Anton

Spain

Kingsley Chidozie Anukam

Nigeria

Virginia Aragon

Spain

Fabrice Armougom

France

Eva Arrebola

Spain

Thomas Atkinson

USA

Matias Attene Ramos

USA

Graeme Attwood

New Zealand

Anna-Marja Aura

Finland

Nicolás Ayub

Argentina

Ramy Karam Aziz

Egypt

Cristina Bacci

Italy

Fabio Bagnoli

Italy

Mario Baigorí

Argentina

Pavol Bakoss

Slovakia

Jan Balzarini

Belgium

Takeshi Bamba

Japan

Silvia Yumi Bando

Brazil

Julia Bandow

Germany

Ravi Barabote

USA

Andrew Barnes

Australia

Francisco Barona-Gomez

Mexico

Rodolphe Barrangou

USA

Munira Basrai

USA

Joyoti Basu

India

Maximilian Baumann

Germany

Andreas Baumler

USA

Nicolas Bayan

France

Doerte Becher

Germany

Georgios Belibasakis

Switzerland

Clara Belzer

Netherlands

Melika Ben Ahmed

Tunisia

Anna Benedetti

Italy

Beatrice Bercot

France

Frederic Bertels

Switzerland

Francesco Berti

Italy

Stefan Bertilsson

Sweden

Michael Berton

USA

Rupak Bhadra

India

Raj Bhatnagar

India

Tomasz Bielecki

Poland

Franck Biet

France

Sarah Bigot

France

Elisabeth Bik

USA

Raja Biswas

India

Pat Blackall

Australia

Giuseppe Blaiotta

Italy

Jessica Blair

UK

Carlos Blanco

France

Jorge Blanco

Spain

Anne Blanc-Potard

France

Steven Blanke

USA

Thomas Bley

Germany

Patrick Boerlin

Canada

Pierre Bogaerts

Belgium

Blaise Boles

USA

Albert Bolhuis

UK

Rémy Bonnin

France

Valeria Bortolaia

Denmark

Nagihan Bostanci

Switzerland

Alfred Botha

South Africa

German Bou

Spain

Yan Boucher

Canada

David Bourne

Australia

Jessica Boyd

Nigeria

Susan Boyle-Vavra

USA

Annabel Braem

Belgium

Fabio Braga

Brazil

Gilberto Braga

Brazil

Olivier Braissant

Switzerland

Catherine Branger

France

Charles Brenner

USA

Stéphane Bretagne

France

Christopher Brigham

USA

Julien Brillard

France

David Brockwell

UK

Ditlev Egeskov Brodersen

Denmark

Barbara M Broeker

Germany

Michael Bromley

UK

Roland Brosch

France

Volker Brozel

USA

Emanuela Bruscia

USA

Thomas Brüser

Germany

Jessica Bryant

USA

Carmen Buchrieser

France

Irene Burckhardt

Germany

Kaye Burgess

Ireland

Lori L. Burrows

Canada

Jeremy Burton

Canada

Ulrich Busch

Germany

Karen Bush

USA

Patrick Butaye

Belgium

Ludovica Buttó

Ireland

Nicholas Butzin

USA

Larry Byers

USA

Barbara Byrne

USA

Esperanza Cabrera

Philippines

Rob Cain

UK

Christopher Marlowe Caipang

Japan

António Calado

Portugal

Cécile Callon

France

Stefano Calo'

Italy

Theodora Calogeropoulou

Greece

Miguel Camara

UK

Andrew Cameron

Canada

Floriana Campanile

Italy

Kenneth Campellone

USA

Stephane Canaan

France

Austin Cantor

USA

James Carey

Taiwan

Steve Carlson

USA

Oliana Carnevali

Italy

David Caron

USA

Cecilia Godoy Carvalhaes

Brazil

Eric Cascales

France

Mike Cashel

USA

Beatrice Casini

Italy

Daniele Casini

Italy

Sherwood Casjens

USA

Lucia Cavalca

Italy

Edna Cavallini Sanches

Brazil

Daniela Ceccarelli

USA

E Cerdá-Olmedo

Spain

Stephane Chaillou

France

Florian Chain

France

Parin Chaivisuthangkura

Thailand

Verawat Champreda

Thailand

Dinesh Chandel

USA

Josephine Chandler

USA

Michael Chandler

France

Ramaswamy Chandrashekar

USA

Gwong-Jen Chang

USA

Perng-Kuang Chang

USA

Yapeng Chao

China

Trevor Charles

Canada

Emmanuelle Charpentier

Sweden

Arnaud Chastanet

France

Roy Chaudhuri

UK

Franck Chauvat

France

Luis Chavez De Paz

Sweden

Huizhong Chen

USA

Xian-Ming Chen

USA

Ying-Lien Chen

Taiwan

Yuansha Chen

USA

Hai-Ping Cheng

USA

Ameur Cherif

Tunisia

Michael Chikindas

USA

Luciane Chimetto

Brazil

Ludmila Chistoserdova

USA

David Chitwood

USA

Karuna Chourey

USA

Rukhsana Chowdhury

India

Henry Choy

USA

Duen-Yau Chuang

Taiwan

Mee-Len Chye

Hong Kong

Nick Cianciotto

USA

Maria Ciaramella

Italy

Judyta Cielecka-Piontek

Poland

Oana Ciofu

Denmark

Clifford Clark

Canada

Thomas Clarke

UK

Jill Clarridge

USA

Erika Claud

USA

Thomas Clavel

Germany

Muriel Cocaign-Bousquet

France

Maria Carmen Collado

Spain

Justine Collier

Switzerland

Cynthia Collins

USA

Mauro M. Colombo

Italy

Fabian Commichau

Germany

Ciaran Condon

France

Brian Coombes

Canada

Pierre Cornelis

Belgium

Naima Gisela Cortes-Perez

France

Don Cowan

South Africa

Aurelie Crabbe

USA

Simon Croft

UK

Elena Crotti

Italy

Anne-Marie Crutz-Le Coq

France

Jaime Cubero

Spain

Roy Curtiss

USA

Tim Cushnie

Thailand

Fouad Dabboussi

Lebanon

Tal Dagan

Germany

Will Dampier

USA

Tanja Dapa

Italy

Ana Lucia Darini

UK

Richard Darveau

USA

Phani Das

USA

Angus Dawe

USA

Michael Day

USA

Frank Dazzo

USA

Frans J. De Bruijn

France

Jeroen De Buck

Canada

Marcus De Goffau

Netherlands

Roberto De Guzman

USA

Christian De La Fe

Spain

Maria Das Graças De Luna

Brazil

Donatella De Pascale

Italy

Hilde De Reuse

France

Olga De Smidt

South Africa

Paul De Vos

Netherlands

Kirk Deitsch

USA

Susana Delgado

Spain

Giovanni Delogu

Italy

Erick Denamur

France

Prashant Desai

USA

Pieter Deschaght

Belgium

Eric Déziel

Canada

Subramanian Dhandayuthapani

USA

Giovanni Di Bonaventura

Italy

Pier Paolo Di Nocera

Italy

Dzung Diep

Norway

Steve Diggle

UK

Elizabeth Dinsdale

USA

Ulrich Dobrindt

Germany

Yohei Doi

USA

Stefano Donadio

Italy

Janet Donaldson

USA

Tao Dong

Canada

Angela Douglas

UK

Xavier Dousset

France

Chrysostomos Dovas

Greece

Max Dow

Ireland

William Dowhan

USA

Michel Drancourt

France

Adam Driks

USA

Zhu Du

China

Zongmin Du

China

Gyanendra P. Dubey

Israel

Eugenie Dubnau

USA

Alain Dufour

France

Roger Dumke

Germany

Maud Dumoux

UK

Gary Dunny

USA

Sylvain Durand

France

Jose Echenique

Argentina

Dale Edmondson

USA

Susan Egan

USA

Thomas Egli

Switzerland

Mitsuru Eguchi

Japan

Sigrun Eick

Switzerland

Alexander Eiler

Sweden

Tony Eissa

USA

Karin Elberse

Netherlands

Marie Elliot

Canada

Akihito Endo

Finland

Danilo Ercolini

Italy

Gisela F Erf

USA

Woldaregay Erku Abegaz

Ethiopia

Robert Ernst

USA

Clara Espitia

Mexico

Jaime Esteban

Spain

Manuel Etienne

France

Chad Euler

USA

Thaddeus Ezeji

USA

Anbin Ezhilan

Cambodia

David Ezra

Israel

Hiroshi Ezura

Japan

Paul Facey

UK

Alan Fahey

Ireland

Maria Faleiro

Portugal

Firouzeh Fallahi

Canada

Weihuan Fang

China

Sabeena Farvin

Denmark

Guido Favia

Italy

Peter Feng

USA

Tom Ferenci

Australia

Henrique Ferreira

Brazil

Aretha Fiebig

USA

Agnes Figueiredo

Brazil

Melanie Filiatrault

USA

Peter Fineran

New Zealand

Vincent Fischetti

USA

Andre Fleissner

Germany

Hansel Fletcher

USA

Antje Flieger

Germany

Ad Fluit

Netherlands

Steven Foley

USA

Jason Folster

USA

William Fonzi

USA

Steven Forst

USA

Konrad Ulrich Förstner

Germany

Jeffrey Foster

USA

Fiona Fouhy

Ireland

Arthur Frampton

USA

M. Pilar Francino

Spain

Jose Franco Da Silveira

Brazil

Laura Franzetti

Italy

Elizabeth G.A. Fredheim

Norway

Stephen Free

USA

Joachim Frey

Switzerland

W. Florian Fricke

USA

Ville-Petri Friman

UK

Teresa Frisan

Sweden

Katsuhiko Fujii

Japan

Takao Fujii

Japan

Yasutaro Fujita

Japan

Chang-Phone Fung

Taiwan

Ricardo Furlan

Argentina

Paolo Gaibani

Italy

Irene Galani

Greece

Cesira Galeotti

Italy

Rodrigo Galhardo

USA

Antonia Gallo

Italy

Han Ming Gan

Malaysia

Pedro Garcia

Spain

Ana L. Garcia-Perez

Spain

Richard Gardner

New Zealand

Victoriano Garre

Spain

Genevieve Garriss

France

Graciela Garrote

Armenia

Patrice Gaurivaud

France

Beilei Ge

USA

Otto Geiger

Mexico

Hans Geiselmann

France

Pierre Genevaux

France

Stephane Genin

France

Joan Geoghegan

Ireland

Nicole Gerardo

USA

Johannes Gescher

Germany

Borbala Gesser

Denmark

Rohit Ghai

Spain

Giovanni Gherardi

Italy

Anuradha Ghosh

USA

Jean-Christophe Giard

France

John Gibbons

USA

Osnat Gillor

Israel

Giorgio Giraffa

Italy

Filipa Godoy-Vitorino

USA

Joao Gomes

Portugal

Fernando Gomez

Spain

Laura Gomez Valero

France

Oscar Gomez-Duarte

USA

Margarita Gomila

Spain

Thially Gonçalves

Brazil

Ursula Gonzales-Barron

Portugal

Angel Gonzalez

Colombia

Bernardo Gonzalez

Chile

Narjol Gonzalez-Escalona

USA

Dolores Gonzalez-Pacanowska

Spain

Venkat Gopalan

USA

Uri Gophna

Israel

David Gordon

Australia

Boris Görke

Austria

Paul Gorry

Australia

Pontus Gourdon

Denmark

Jeffrey Gralnick

USA

Peter Graumann

Germany

Jeff Green

UK

Gustavo Bueno Gregoracci

Brazil

Daniel Grenier

Canada

Scott Grieshaber

USA

Régis Grimaud

France

Elisabeth Grohmann

Germany

Rita Grosch

Germany

Ioannis Gryllos

USA

Josep Guarro

Spain

Thomas Guillard

France

Felizza Gunderson

USA

Robert Gunsalus

USA

Sebastian Günther

Germany

Amita Gupta

India

Reinhard Guthke

Germany

Marcel Gutiérrez-Correa

Peru

José Ángel Gutiérrez-Pabello

Mexico

Olivier Habimana

Ireland

Ted Hackstadt

USA

Ferry Hagen

Netherlands

Elisabeth Haggård

Sweden

Matthias Hahn

Germany

Constantine Haidaris

USA

Ruth Hall

Australia

Otto Haller

Germany

Takashi Hamabata

Japan

Michael Hamblin

USA

Lennart Hammarström

Sweden

Yanping Han

China

Robert Hancock

Canada

Scott Handley

USA

Irene Hanning

USA

Harapan Harapan

Indonesia

Josee Harel

Canada

Freya Harrison

UK

Axel Hartke

France

Alain Hartmann

France

John Hartung

USA

Tadao Hasegawa

Japan

Anna Haukioja

Finland

Susanne Haussler

Germany

Yoichi Hayakawa

Japan

Shelley Haydel

USA

Qigai He

China

Ya-Wen He

China

Yongqun He

USA

Stephan Heeb

UK

Ralf Heermann

Germany

Norton Heise

Brazil

Dennis Helfritch

USA

Eric Hendrickson

USA

Nicholas Heng

New Zealand

Isabel Henriques

Portugal

Mariana Henriques

Portugal

Thomas Henry

France

Michael Hensel

Germany

Paul Hergenrother

USA

Stefan Hertwig

Germany

Claudia Hess

Austria

Kim Heylen

Belgium

Janet Hill

Canada

Daniela Hirata

Brazil

Christoph Hoegenauer

Austria

Achim Hoerauf

Germany

Niels Høiby

Denmark

Matthew Holden

UK

Christina Hölzel

Germany

Takahiro Hosokawa

Japan

Eric Houpt

USA

Yen-Ping Hsueh

USA

Fupin Hu

China

Wenhui Hu

USA

Kaiyao Huang

China

Xiaozhe Huang

USA

Johannes Huebner

Germany

Diarmaid Hughes

Sweden

Kristina Hulten

USA

Hien-Ching Hung

Taiwan

Sabine Hunke

Germany

David Hunstad

USA

Javid Hussain

Oman

Mohamed Husseiny

USA

Geert Huys

Belgium

Kevin Hybiske

USA

Michael F. Hynes

Canada

Adriana Ianieri

Italy

Valerio Iebba

Italy

Savarimuthu Ignacimuthu

India

Aamer Ikram

Pakistan

Iliyan Iliev

USA

Flavia Indrio

Italy

Masayori Inouye

USA

Heribert Insam

Austria

Thomas Inzana

USA

Nicole Iovine

USA

Hon Ip

USA

Lourdes Isaac

Brazil

Suzanne Ishaq

USA

Mohammad Islam

Bangladesh

Elena Ivanova

Australia

Andrew Paul Jackson

UK

Dan Jacobson

South Africa

Cedric Jacqueline

France

Guilhem Janbon

France

Jonathan Jantsch

Germany

Sophie Jarraud

France

Che Ok Jeon

South Korea

Carlos A. Jerez

Chile

Travis Jewett

USA

Yinduo Ji

USA

Rongrong Jiang

Singapore

Paul Johnston

Germany

Kathryn Jones

USA

Ryan Jones

USA

Kieran Jordan

Ireland

Hans Jørgen Lyngs Jørgensen

Denmark

Olivier Joubert

France

Estelle Jumas-Bilak

France

Tae Sung Jung

South Korea

Juan Luis Jurat-Fuentes

USA

Klaus Jürgens

Germany

Praveen Juvvadi

USA

David Kadosh

USA

Fredrik Kahn

Sweden

Michael Kahn

USA

Jessica Kajfasz

USA

Chrysanthi Kalloniati

Greece

Donata Kalthoff

Germany

Susan Kaminskyj

Canada

Biao Kan

China

Ramani Kandasamy

India

Drosos Karageorgopoulos

Greece

Nabil Karah

Norway

Magnus Karlsson

Sweden

Michihiko Kataoka

Japan

Sophia Kathariou

USA

Lee Katz

USA

Michael Kaufman

USA

Kevin Kavanagh

Ireland

Daniel Kearns

USA

David Kelly

UK

Linda Kelly

USA

William Kelly

New Zealand

Jan Keltjens

Netherlands

David Kelvin

Canada

Nemat Keyhani

USA

Yoshitomo Kikuchi

Japan

Dong Wook Kim

South Korea

Amy Kirby

USA

David Kirchman

USA

Viswanath Kiron

Norway

Leif Kirsebom

Sweden

Mitsuo Kishi

Japan

Haruki Kitazawa

Japan

Balaji Kithiganahalli

India

Marlise Klein

USA

Jörg Kleinschmidt

Germany

Laura Klepp

Argentina

Jeanna Klinth

Sweden

Olaf Kniemeyer

Germany

Christine Knox

Australia

Donald Kobayashi

USA

Ali Kocyigit

Turkey

Michio Koide

Japan

Satoshi Koike

Japan

Tadazumi Komiyama

Japan

Michael Konkel

USA

Konstantinos Kormas

Greece

Victoria Korolik

Australia

Akos T Kovacs

Germany

Bryan Krantz

USA

Jens Kreth

USA

Marco Aurelio Krieger

Brazil

Bastiaan Krom

Netherlands

Andrew Kropinski

Canada

Terry Ann Krulwich

USA

Sidney Ksuhner

USA

Masae Kuboniwa

Japan

Ramesh Chander Kuhad

India

Katrin Kuhls

Germany

Andreas Kuhn

UK

Juliane Kühn

Switzerland

Ranjit Kumar

USA

Gotthard Kunze

Germany

Jozef Kur

Poland

Cletus Kurtzman

USA

Rahul Kuver

USA

Patrick Kwan

USA

Maurizio Labbate

Australia

Richard Lamont

USA

Paolo Landini

Italy

Sue Lang

UK

Kerry Laplante

USA

Martin Lappann

Germany

Enrique Lara

Switzerland

Maria Lara-Tejero

USA

Christine Lascols

USA

Jürgen Lassak

Germany

Elena Lasunskaia

Brazil

Mallika Lavania

India

Vladimir Lazarevic

Switzerland

Hervé Le Moual

Canada

Sarah Lebeer

Belgium

Julie Ledford

USA

Leo Leduc

Canada

Byong Lee

Canada

Chia Lee

USA

Duu-Jong Lee

Taiwan

Jean Lee

USA

Michael Lehman

USA

Angelika Lehner

Switzerland

Ana Lúcia Leitão

Portugal

Francisco Lemos

Brazil

Metka Lenassi

Slovenia

Baptiste Leroy

Belgium

Endang Sri Lestari

Indonesia

Johan Leveau

USA

Celine Levesque

Canada

Shawn Lewenza

Qatar

Shawn Lewenza

Canada

Janina Lewis

USA

L. Kevin Lewis

USA

Meng Li

USA

Shanshan Li

USA

Wen-Jun Li

China

Yang Li

USA

Yong-Quan Li

China

Magdalen Lindeberg

USA

Steven Lindow

USA

David Lindsay

USA

Paloma Liras

Spain

Michael Lisby

Denmark

Trevor Lithgow

Australia

Don Liu

USA

Shu-Lin Liu

Canada

Xingzhong Liu

China

Shyh-Ching Lo

USA

Ramiro Logares

Spain

Nikola Loncar

Serbia

Gitte Loozen

Belgium

Daniel Lopez

Germany

Isabel M Lopez-Lara

Mexico

Jaime Lora-Tamayo

Spain

Remy Loris

Belgium

Charles Lovell

USA

Catherine Lozupone

USA

Xiaonan Lu

Canada

Naomi Lucchi

USA

Vicki Luna

USA

Agnese Lupo

Switzerland

Sara Lustigman

USA

Denis Lynn

Canada

Xuejun Ma

China

Juliette Madan

USA

Veerle Maervoet

Belgium

Tibor Magyar

Hungary

Rehab Mahmoud Abd El-Baky

Egypt

Volker Mai

USA

Tomas Maira-Litran

USA

Tim Maisch

Germany

Judith Choi Wo Mak

China

Iran Malavazi

Brazil

Riccardo Manganelli

Italy

Panchanathan Manivasagan

South Korea

Matteo Marangon

Australia

Gregory Marczynski

Canada

Imma Margarit

Italy

Miles Markus

South Africa

Terence L. Marsh

USA

Rocio Martin

Netherlands

Vincent Martin

Canada

Ana Belen Martin Cuadrado

Spain

Rebeca Martin Rosique

France

Jose Martinez

Spain

Luis Martinez

USA

Manuel Martinez Garcia

Spain

Flaviano Martins

Brazil

Juergen Marxsen

Germany

Aleksander Masny

Poland

Kevin Mason

USA

Paola Mastrantonio

Italy

Laurence Mathieu

France

Ivan Matic

France

Yu Matsuura

Japan

Shigenobu Matsuzaki

Japan

Michael Matthias

USA

Heather Maughan

Canada

Max Maurin

France

Patrick Mavingui

France

Thithiwat May

USA

Luca Mazzon

Italy

J Vaun Mcarthur

USA

Alex Mccarthy

UK

Christine Mccartney

UK

Brendan Mcconkey

Canada

Sheena Mcgowan

Australia

Robert Mclean

USA

Patricia Mcmanus

USA

Alan Mcnally

UK

Joseph Mcphee

Canada

Francis Megraud

France

Samina Mehnaz

Pakistan

Marjolein Meijerink

Netherlands

Markus Meissner

UK

Antonella Mencacci

Italy

Vitor Mendes

UK

Maria Jose Mendes Giannini

Brazil

Dominique Mengin-Lecreulx

France

Jeffrey Mercante

USA

D. Scott Merrell

USA

Stephen Michell

UK

Tore Midtvedt

Sweden

Marika Mikelsaar

Estonia

Catarina Milheiriço

Portugal

Jennifer Miller

USA

William Miller

USA

David Mills

USA

M Miragaia

Portugal

Nagendra Mishra

USA

Prakash Mishra

India

Tohru Miyoshi-Akiyama

Japan

Stefano Mocali

Italy

Ralf Moeller

Germany

Vijayanand Suryakant Moholkar

India

Sylvain Moineau

Canada

Anne Moir

UK

Dominik Mojzita

Finland

Douwe Molenaar

Netherlands

Vicente Monedero

Spain

Clara Monferran

Argentina

Dolly Montoya

Colombia

Hector Morbidoni

Argentina

Gabriel Moreno-Hagelsieb

Canada

Hirotada Mori

Japan

Shunsuke Mori

Japan

Melanie Mormile

USA

Alison Morris

USA

Paul Morris

USA

Charles Oliver Morton

Australia

Paul Moundipa

Cameroon

Takafumi Mukaihara

Japan

Sunil Mukherjee

India

Etienne Muller

South Africa

Matthew Mulvey

USA

Andrey Mulyukin

Russian Federation

Carmen Munoz-Almagro

Spain

Madhaiyan Munusamy

Singapore

Barbara E. Murray

USA

Ewan Murray

UK

Heath Murray

UK

Sean Murray

USA

Kim Musser

USA

Alessandro Muzzi

Italy

Turaya Naas

Canada

Kentaro Nagamine

Japan

Neelakanta Rao Nagireddy

USA

Ravinder Nagpal

Japan

Moon Nahm

USA

Celso Vataru Nakamura

Brazil

Tsutomu Nakamura

Japan

Amine Namouchi

France

Beiyan Nan

USA

Bindu Nanduri

USA

Ramakrishna Nannapaneni

USA

Christian Napoli

Italy

Rodrigo Nascimento

Brazil

Gerardo Nava

USA

Thulile Ndlovu

South Africa

Daniel Neafsey

USA

Natasha Nesbitt

USA

Heinrich Neubauer

Germany

Ming Ni

China

Robin Nicholas

UK

Allen Nicholson

USA

Cheryl Nickerson

USA

Ole Lerberg Nielsen

Denmark

Kendra Nightingale

USA

Reindert Nijland

Netherlands

Anastasia Nijnik

Canada

Vladyslav Nikolayevskyy

UK

Graeme Robert Nimmo

Australia

Michele Nishiguchi

USA

Andreas Nocker

UK

Juan Nogales

Spain

Nobuhiko Nomura

Japan

David Norman

USA

Harald Nothaft

Canada

Nico Nouwen

France

Angela Novais

Portugal

Mairi Noverr

USA

Richard Novick

USA

Forough Leila Nowrouzian

Sweden

Ulrich Nübel

Germany

Armel Herve Nwabo Kamdje

Cameroon

Tuula Nyman

Finland

Sonja Oberbeckmann

Germany

Lene Oddershede

Denmark

Sarah O'Flaherty

USA

James O'Gara

Ireland

David Ogbolu

Nigeria

Hyun-Myung Oh

South Korea

Anna Oksaharju

Finland

Fabiano Oliveira

USA

Maria Leonor Oliveira

Brazil

Andrew Olkowski

Canada

Eoghan O'Neill

Ireland

Onya Opota

Switzerland

Aharon Oren

Israel

Carlos Osorio

Spain

Mark Ott

USA

Andreas Otto

Germany

Ernesto Oviedo-Orta

Italy

Karoly Pal

Hungary

Utpal Pal

USA

Oleg Paliy

USA

Giuseppe Palladino

USA

Lucia Pallecchi

Italy

Mohan Pammi

USA

Rekha Panchal

USA

Barbara Papadopoulou

Canada

Seraphim Papanikolaou

Greece

Kai Papenfort

Germany

Tanya Parish

UK

Chankyu Park

South Korea

Craig Parker

USA

Griffith Parks

USA

Volkmar Passoth

Sweden

Victoria Pastor

Switzerland

Pradip Patel

France

Silvia Patruica

Romania

Cheryl Patten

Canada

Katharina Pawlowski

Sweden

Sophie Payot

France

Raúl Pedraza

Argentina

Trisha Peel

Australia

Stanislav Pepeliaev

Czech Republic

Manuela Pereira

Portugal

Sabine Pereyre

France

Danilo Perez

Spain

Silvia Perotto

Italy

Michel Perreau

France

Marko Pesu

Finland

Fabienne Petit

France

Marie-Agnès Petit

France

Stacy Pfaller

USA

Yvonne Pfeifer

Germany

Davies Mubika Pfukenyi

Zimbabwe

Bodo Philipp

Germany

Renata Picao

Brazil

Harald Pichler

Austria

Gerhard Pietersen

South Africa

Martin Pilhofer

USA

William Pinchak

USA

Mariana Pinho

Portugal

Ines Pinto

USA

Jaume Pinyol

Spain

Laurici Pires

Brazil

Jean-Paul Pirnay

Belgium

Aida Pitarch

Spain

Andre Pitondo-Silva

Brazil

Johann Pitout

Canada

Mariagrazia Pizza

Italy

Marie-Cécile Ploy

France

Rainer Podschun

Germany

Benoit Polack

France

Andrey Pomerantsev

USA

Monica Ponder

USA

Sven Poppert

Germany

Yannick Poquet

France

Andrea Porras-Alfaro

USA

Klaas Pos

Germany

Jan Potempa

USA

Spyros Pournaras

Greece

Shane Powell

Australia

Renato Prates

Brazil

Chet Price

USA

Karin Pritsch

Germany

Birgit Pruess

USA

Olivier Pruvost

France

Anna Psaroulaki

Greece

Thomas Pufe

Germany

Guanghai Qi

China

Enrique Quesada Moraga

Spain

Gerry Quinn

Ireland

Janet Quinn

UK

Balazs Rada

USA

Robbie Rae

Germany

Gireesh Rajashekara

USA

Mirjana Rajilic-Stojanovic

Serbia

Gordon Ramage

UK

Thandavarayan Ramamurthy

India

Jesus Ramos

Brazil

Ashish Ranjan

USA

Rino Rappuoli

Italy

James Rasheed

USA

Geertrui Rasschaert

Belgium

Bala Rathinasabapathi

USA

Raul Raya

Argentina

Ben Raymond

UK

Lúcia Rebello Dillenburg

Brazil

Mario Recker

UK

Hans Rediers

Belgium

Cath Rees

UK

Abdul Rehman

Pakistan

Cornelia Reimmann

Switzerland

Bin Ren

Australia

Karin Rengefors

Sweden

Sandra Reuter

UK

Linda Rhodes

USA

Olin Rhodes

USA

Laurie Richardson

USA

Katharina Riedel

Germany

Kristian Riesbeck

Sweden

Daniel Rigden

UK

Margaret Riley

USA

Andrea Rinaldi

Italy

Daniela Rinaudo

Italy

Rafael Rivilla

Spain

Hyeon Su Ro

South Korea

Adam Roberts

UK

Roy Robins-Browne

Australia

Ashley Robinson

USA

Eric Roche

USA

Dan Rockey

USA

Francisco Rodriguez-Valera

Spain

Geraint Rogers

UK

James Rogers

USA

Forest Rohwer

USA

Sara Romano

France

Stefan Roos

Sweden

Bruce Rosa

USA

Ninfa Rosas-García

Mexico

Adriana Rosato

USA

Ramon Rosselló-Móra

Spain

Maddalena Rossi

Italy

Gian Maria Rossolini

Italy

Michael Rother

Germany

Bart Roucourt

Belgium

Denis Roy

Canada

Edward Ruby

USA

Knut Rudi

Norway

Karen Rudolph

USA

Victor Pmg Rutten

Netherlands

Robert Ryan

UK

Ivan Rychlik

Czech Republic

Patrik Rydén

Sweden

Sangryeol Ryu

South Korea

Sophie Sable

France

Ewa Sadowy

Poland

Yolanda Sáenz

Spain

Hany Sahly

Germany

Milton Saier

USA

Raquel Sa-Leao

Portugal

Jarkko Salojärvi

Finland

Anne Salonen

Finland

John Samelis

Greece

Josep Samitier

South Africa

Sergio Sanchez

Mexico

Félix J Sangari

Spain

Marina Santic

Croatia

Joanne Santini

UK

Angelo Santino

Italy

Frank Sargent

UK

Alain Sarniguet

France

Inka Sastalla

USA

John-Demian Sauer

USA

Hendrik Schaefer

UK

Dirk-Jan Scheffers

Netherlands

Catherine H. Schein

USA

Andreas Schlüter

Germany

Imke Schroeder

USA

Sven Schuchardt

Germany

Stephanie Schuller

UK

Clarissa Schwab

Austria

William Schwan

USA

Stefan Schwarz

Germany

Andreas Schwiertz

Germany

Ian Scott

New Zealand

Ondrej Sedo

Czech Republic

Jessica Seeliger

USA

Shamala Devi Sekaran

Malaysia

Joseph Selvin

India

Jayampath Seneviratne

Hong Kong

Hidenobu Senpuku

Japan

Ethan Settembre

USA

Joao Setubal

Brazil

Joao Setubal

USA

Anja Seubert

Italy

Konstantin Severinov

USA

Mohammad Reza Shakibaie

Iran

Wu Shangong

China

Nathan Shankar

USA

Rishi Shanker

India

Xuzhuang Shen

China

Zhangqi Shen

USA

Philip Sherman

Canada

Takeshi Shimosato

Japan

Annie Shrestha

Canada

Sanjay Shukla

USA

Jatinder Sidhu

Australia

Lotta Siira

Finland

Mark Silby

USA

Sanna Sillankorva

Portugal

Lúcia C. Simões

Portugal

Philippe Simoneau

France

Ankit Singla

Japan

Sunil Sirohi

India

Aleksandra Sklodowska

Poland

Barton Slatko

USA

Daniel Sloan

USA

David Smajs

Czech Republic

Anna Smed-Sorensen

Sweden

Cindy Smith

Ireland

Duncan R. Smith

Thailand

Peter Solomon

Australia

Greg Somerville

USA

Leonardo Sorci

Italy

Irma Soria-Mercado

Mexico

Vanessa Sperandio

USA

Pietro Speziale

Italy

Lisa D. Sprague

Germany

Emma Sproston

Canada

Padma Srikanth

India

Lola Stamm

USA

Nicola Stanley-Wall

UK

Susan Steenbergen

USA

Paola Stefanelli

Italy

Daniel Stein

USA

Lilian Stepehen

Nigeria

Scott Stibitz

USA

Maria Silvina Stietz

Argentina

Tim Stinear

Australia

Alain Stintzi

Canada

Stephen Stockdale

Ireland

Beatriz Stolf

Brazil

Joanne Stubbe

USA

Juan Suarez

Spain

Elankumaran Subbiah

USA

Agathe Subtil

France

Fengjie Sun

USA

Jianbo Sun

USA

Sheng Sun

USA

George Sundin

USA

Yasuhiro Suzuki

USA

Liselott Svensson-Stadler

Sweden

Steven Szczepanek

USA

Carlos Taborda

Brazil

Daniel Tadesse

USA

John Tagg

New Zealand

Seiichi Taguchi

Japan

Masahiro Takeo

Japan

Lionel Tan

UK

Valter Tandoi

Italy

Yi-Wei Tang

USA

Shuji Tani

Japan

Tokio Tani

Japan

Fernando Tavares

Portugal

Mark Taylor

UK

Neus Teixidó

Spain

Christoph Tellenbach

Switzerland

Max Teplitski

USA

Guillaume Tetreau

USA

Dirk Theegarten

Germany

Francois Thiaucourt

France

Anne Thierry

France

Linda Thomashow

USA

Fabiano Thompson

Brazil

Michael Thon

Spain

Kai Thormann

Germany

Chang Fu Tian

China

Petra Tielen

Germany

Salme Timmusk

Sweden

Masanori Tohno

Japan

Janetta Top

Netherlands

Carmen Torres

Spain

Eva Torres

Spain

Makrina Totsika

Australia

Patricia Totten

UK

Rosalia Trias

France

Gena Tribble

USA

James Triccas

Australia

Paal Trosvik

Norway

Lisbeth Truelstrup Hansen

Canada

Apichai Tuanyok

USA

Jane Turton

UK

Jaya Tyagi

India

Milton Typas

Greece

Søren Uldum

Denmark

Gottfried Unden

Germany

David Ussery

USA

Fatemeh Vahedi

Iran

Florence Val

France

Raphael Valdivia

USA

Henny Van Der Mei

Netherlands

Menno Van Der Voort

Netherlands

Jan Maarten Van Dijl

Netherlands

Eibert Van Engelen

Netherlands

Martijn Van Hemert

Netherlands

Steven Van Lanen

USA

Jos Van Strijp

Netherlands

Jos Vanderleyden

Belgium

Pierre Vaudaux

Switzerland

Jose A. Vazquez

Spain

Vittorio Venturi

Italy

Bjorn Vergauwen

Belgium

Andre Vermeglio

France

Kevin J. Verstrepen

Belgium

Miguel Vicente

Spain

Anne Vidaver

USA

Beatrice Vitali

Italy

Kerstin Voelz

UK

Amy Vogler

USA

Ingemar Von Ossowski

Finland

Claes Von Wachenfeldt

Sweden

Atte Von Wright

Finland

Julia Vorholt

Switzerland

Michiel Vos

UK

Romé Voulhoux

France

Rolf Wagner

Germany

Mark Wainwright

UK

Alan Walker

UK

Graham Walker

USA

Jens Walter

USA

Gejiao Wang

China

Guanghua Wang

China

Hengzhuang Wang

Denmark

Junwei Wang

China

Minggui Wang

China

Nian Wang

USA

Qian-Qiu Wang

China

Xiaoyu Wang

USA

Yi Wang

USA

Len Ward

Canada

Koichi Watanabe

Japan

Rory Watt

Hong Kong

Stefan Weckx

Belgium

Hong Wei

China

Christopher Weidenmaier

Germany

Francois-Xavier Weill

France

David Weiss

USA

Guido Werner

Germany

Philip Wescombe

New Zealand

Michael Wessels

USA

Emily Whiston

USA

Emmanuel Wicker

Reunion

Micael Widerstrom

Sweden

Kinga Wieczorek

Poland

Mark Willcox

Australia

Anne Willems

Belgium

Linette Willemsen

Netherlands

Thomas Wittum

USA

Patrick Cy Woo

Hong Kong

Martin Woodward

UK

Jonathan Worrall

UK

Brendan Wren

UK

Congming Wu

China

Shih-Hsiung Wu

Taiwan

Qiaobin Xiao

USA

Tian Xiao

China

Zhixun Xie

China

Huaxi Xu

China

Jianguo Xu

China

Jiannong Xu

USA

Jianping Xu

Canada

Xiang Xu

USA

Xudong Xu

China

Zhaohui Xu

USA

Zhenbo Xu

China

Chaoyang Xue

USA

Kai Xue

USA

Hideki Yamaji

Japan

Seiya Yamayoshi

Japan

Frank Yang

USA

Kathy Yang

USA

Ruifu Yang

China

Shihui Yang

USA

Changyun Ye

China

Wu Yimou

China

Hideo Yonezawa

Japan

Pauline Yoong

USA

Yuko Yoshikawa

Japan

Chris Yost

Canada

Jung-Won Youn

Germany

J Peter Young

UK

Kevin Young

USA

Ming Yuan

China

André Zapun

France

Oscar Zaragoza

Spain

Hassan Zaraket

USA

Raffaele Zarrilli

Italy

Anton Zavialov

Finland

Darja Zgur-Bertok

Slovenia

Cheng-Cai Zhang

France

Haili Zhang

USA

Qijing Zhang

USA

Wei Zhang

USA

Wenbo Zhang

USA

Xue-Xian Zhang

New Zealand

Jindong Zhao

China

Mujun Zhao

China

Youfu Zhao

USA

Huachen Zhu

Hong Kong

Yongqun Zhu

China

Wilma Ziebuhr

Germany

Andrea Zille

Portugal

Karin Zitterl-Eglseer

Austria

James Zlosnik

Canada

Shaolan Zou

China

Tiago Zucchi

Brazil

Daniela Zuehlke

Germany

Daniel Zurawski

USA

Ludek Zurek

USA

